# Supplementary figures and images for: Iron availability and oxygen tension regulate the Yersinia Ysc type III secretion system to enable disseminated infection
Source: PLoS Pathog. 2019 Dec 23;15(12):e1008001. doi: 10.1371/journal.ppat.1008001 (PMC6946166; doi:10.1371/journal.ppat.1008001)

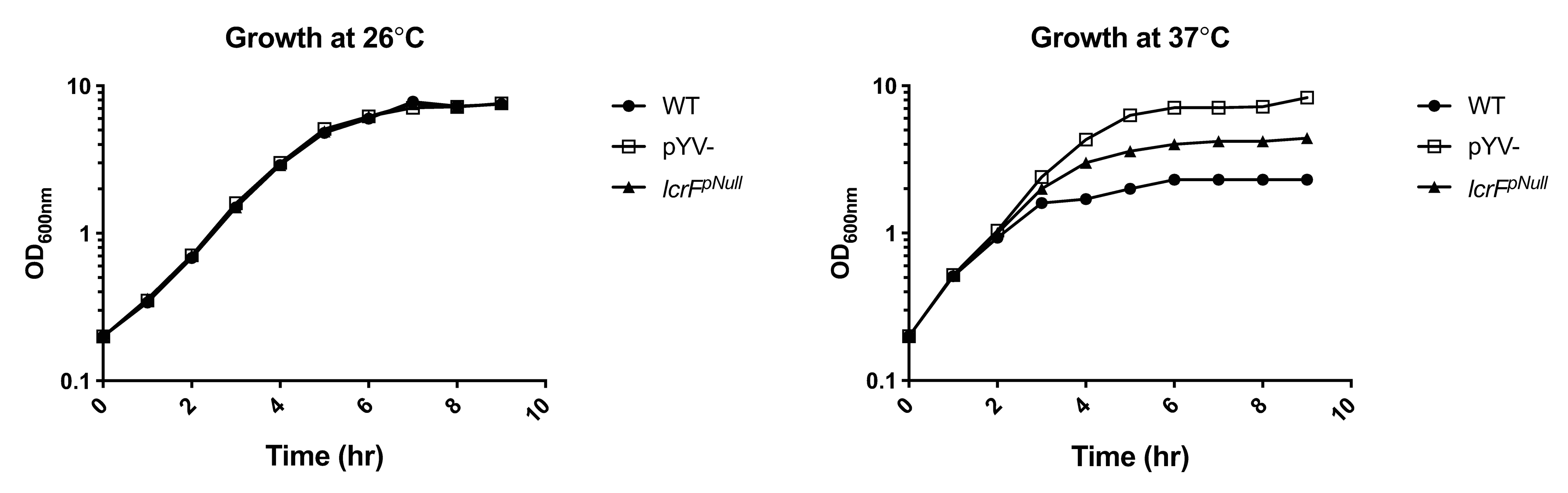

Supplement: S1 Fig — Overnight bacterial cultures were subcultured into fresh M9 media containing iron and grown under aerobic condtions for 9 hrs at either 26°C or 37°C. OD600 was measured every hour. Note that the pYV- mutant lacking all T3SS genes does not undergo the normal growth arrest seen in wildtype Yersinia following T3SS induction at 37°C [63]. (TIF) [file ppat.1008001.s001.tif]

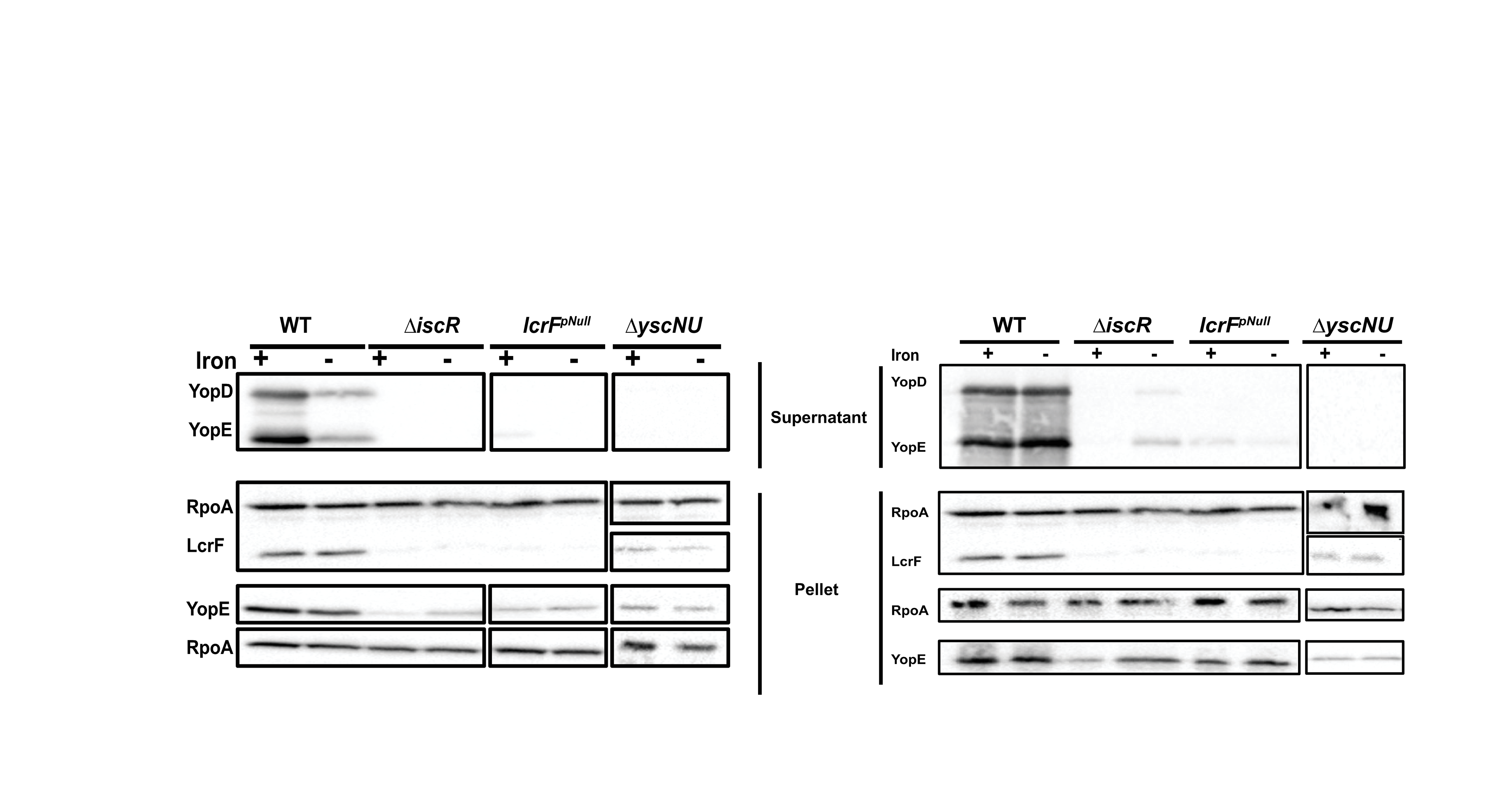

Supplement: S2 Fig — Two additional independent replicates of the results shown in Fig 3 are provided. Iron-limited Y. pseudotuberculosis were induced for the T3SS under aerobic iron-replete (+Fe) or iron-limited (-Fe) conditions as described in Fig 3 and assayed for proteins by Western blots. Top panels, supernatant. Bottom panels, cell pellet. (TIF) [file ppat.1008001.s002.tif]

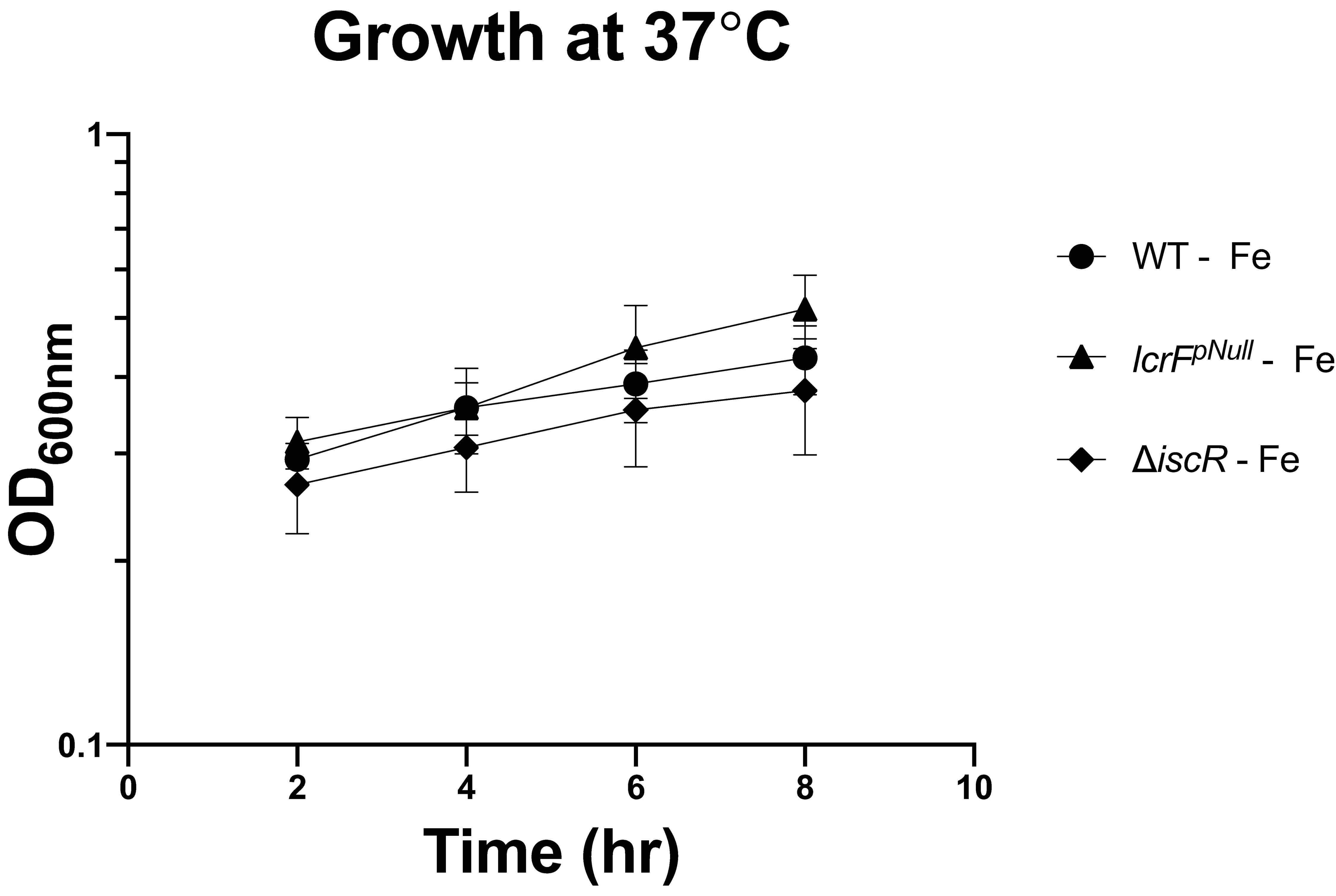

Supplement: S3 Fig — Y. pseudotuberculosis strains were iron starved under anaerobic conditions and OD600 was measured every hour after shifting to 37°C. Data shown represent the average of three independent experiments. (TIF) [file ppat.1008001.s003.tif]

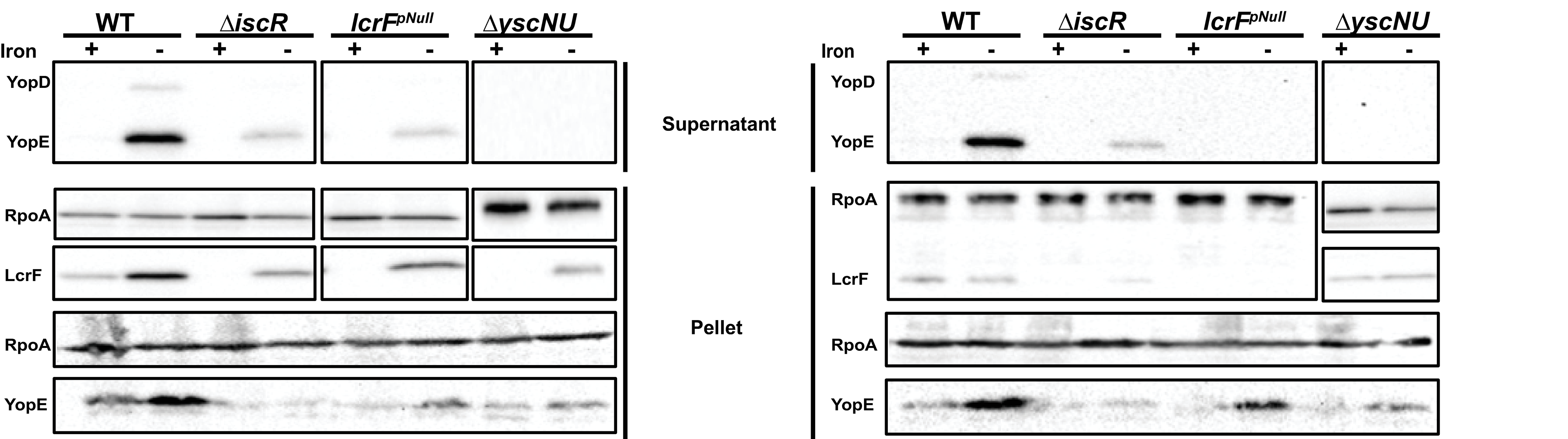

Supplement: S4 Fig — Y. pseudotuberculosis was iron starved and grown for 12 hours in the absence of oxygen prior to inducing the T3SS by shifting to 37°C, as in Fig 4. Two additional independent replicates are shown. Top panels, supernatant. Bottom panels, cell pellet. (TIF) [file ppat.1008001.s004.tif]

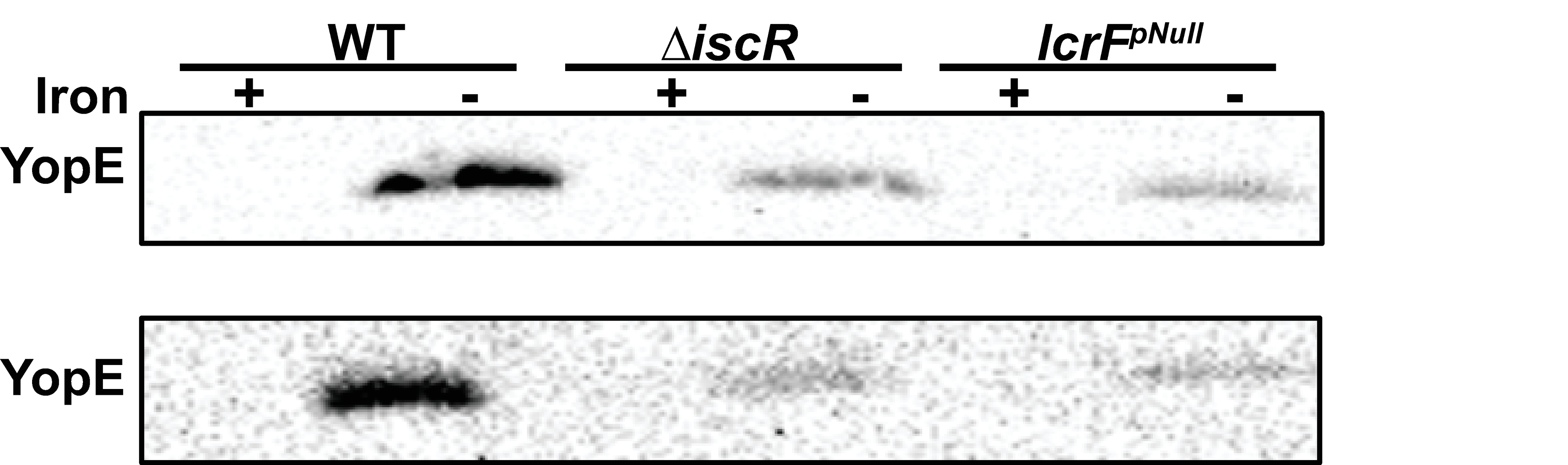

Supplement: S5 Fig — Y. pseudotuberculosis was iron starved and grown for only four hours in the absence of oxygen prior to inducing the T3SS by shifting to 37°C. Secreted proteins were precipitated with TCA and analyzed by Western blot. Two independent experiments are shown. (TIF) [file ppat.1008001.s005.tif]

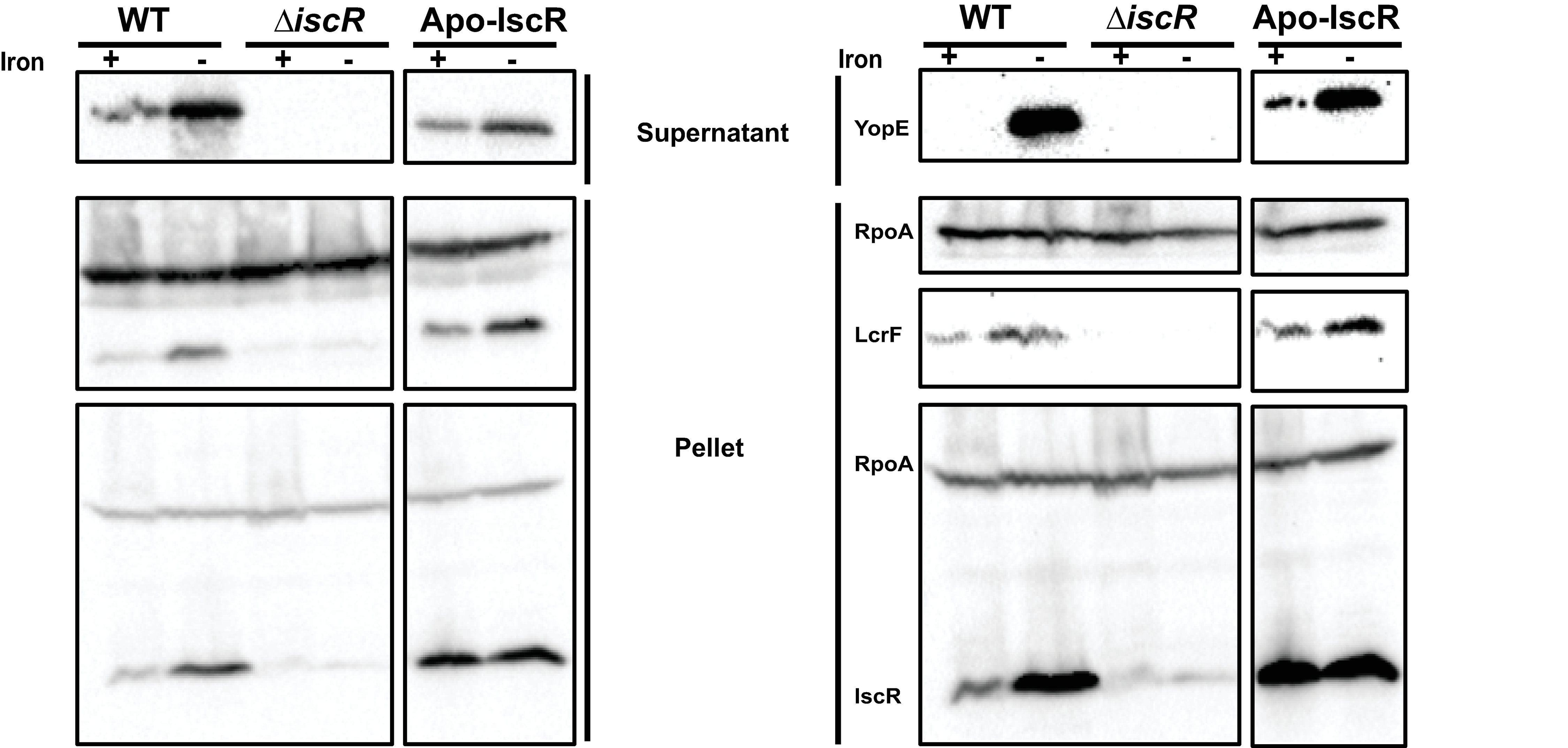

Supplement: S6 Fig — Iron starved Y. pseudotuberculosis was grown under anaerobic conditions in M9 supplemented with nitrate and mannitol instead of glucose to support anaerobic respiration. Cultures were then shifted to 37°C and both secreted and intracellular proteins were analyzed by Western blot, as in Fig 5. Two additional independent replicates are shown. Top panels, supernatant. Bottom panels, cell pellet. (TIF) [file ppat.1008001.s006.tif]
