# Supplementary material for: Iron availability and oxygen tension regulate the Yersinia Ysc type III secretion system to enable disseminated infection
Source: PLoS Pathog. 2019 Dec 23;15(12):e1008001. doi: 10.1371/journal.ppat.1008001 (PMC6946166; doi:10.1371/journal.ppat.1008001)
Supplement: Attachment — Submitted filename: Hooker-Romero rebuttal corrected.docx [file ppat.1008001.s007.docx]

**Response to reviewer comments for Hooker-Romero et al**

Reviewer #1: Additional Experiments:
• The experiment with Y. pestis (Figure 8) was performed under different conditions (+/- Ca2+) to induce the T3SS; an additional experiment with Y. pseudotuberculosis performed in the same way is needed to directly compare between Y. pestis and Y. pseudotuberculosis.

We have added a new experiment showing *Y. pseudotuberculosis* type III secretion under the same conditions in the revised Figure 8.

• The Apo-IscR mutant was tested under both iron replete and iron limited conditions (line 253) for the anaerobic respiration conditions (Figure 5), but was not tested under iron limited conditions in Figure 6. Was there a reason this wasn’t included? I think this is needed to support the connection between IscR levels and T3SS induction (lines 266-267), since the Apo-IscR mutant is also lysing under anaerobic conditions (lines 260-263).

There are no iron depleted conditions in Figure 6. Figure 6 only shows *Yersinia* cultures that have never been iron depleted subjected to anaerobic conditions (same conditions as in Figure 2). We have included a statement in the revised manuscript clarifying that the conditions used for Figures 2 and 6 are identical (line 694).

The Apo-IscR mutant was included in Figure 5, which shows *Yersinia* cultures starved of iron and then incubated under anaerobic iron replete or depleted conditions. The Apo-IscR mutant was not shown under aerobic conditions, as we previously showed that this mutant has a proton motive force defect in the presence of oxygen, and therefore cannot secrete Yops (Miller et al 2014 *PLoS Pathogens*).

While the Apo-IscR mutant showed a trace amount of lysis under the anaerobic, non-iron starved conditions in Figure 6, this should not affect detection of cytoplasmic IscR or LcrF. This is discussed in lines 281-288.

Major Concerns:
• It was unclear what is known about the requirements for T3SS expression during enteropathogenic Yersinia infection. The abstract suggests that T3SS expression is required for dissemination, but not required for colonization of the intestine; is there a requirement for expression in Peyer’s patches? The results presented here suggest expression is only required for dissemination, was this a novel finding? Or is this consistent with published literature? A discussion of previous studies with strains lacking the virulence plasmid, or lacking T3SS function specifically, would help put this study in context.

The *Yersinia* T3SS contributes to colonization of the Peyer’s patches (PP) and mesenteric lymph nodes (MLN) early during infection (2 days), but by day 5, absence of a functional T3SS does not appear to affect colonization of these organs. In contrast, there is an absolute requirement for the T3SS in colonization of the spleen and liver. Similarly, we find a severe hepatosplenic colonization defect for the *lcrF*^pNull^ mutant, but no significant defect by 5 days post-inoculation for the PP and MLNs. We have clarified this in the revised discussion (lines 375-380).

• If both Y. pseudotuberculosis and Y. pestis utilize a similar mechanism for T3SS expression, then does this suggest T3SS expression is most important in a niche they both occupy? Such as lymph nodes or deep tissues? More discussion should be included to consider whether T3SS expression is needed within distinct tissues, such as mesenteric lymph nodes. Do these results suggest Y. pestis is still adapted to intestinal tissues, and may not be regulating T3SS expression as efficiently as it could?

*Y. pestis* in the rat bubo experiences iron limitation and utilizes mainly anaerobic metabolism 2-3 days post-inoculation (Sebbane et al 2016 *PNAS*), similar to what *Y. pseudotuberculosis* experiences in the Peyer's patches two days post-inoculation (Nuss et al 2016 *PNAS*). **Therefore, in terms of iron and oxygen, *Y. pestis* and *Y. pseudotuberculosis* seem to experience their lymph node niches similarly.** At these early time points, the T3SS contributes to lymph node colonization for both pathogens. However, specifically for *Y. pseudotuberculosis*, the T3SS isn’t required for later colonization of gut-associated lymph tissue (5 days post-inoculation such as in our study). Furthermore, *Y. pseudotuberculosis* in lymph nodes do not go on to successfully seed deeper tissues in healthy mice (see below). **The T3SS is essential for both *Y. pseudotuberculosis* and *Y. pestis* to colonize the spleen and the liver.** In agreement with this, we find that IscR control of the T3SS is essential for *Y. pseudotuberculosis* infection of the spleen and liver, but not gut-associated lymph tissues, 5 days post-inoculation (Fig 8).

*Y. pseudotuberculosis* colonization of lymph tissue does not seem to be a conduit for spread to the spleen and liver (Barnes et al 2006 *JEM*). Instead, a replicating pool of *Y. pseudotuberculosis* in the intestinal lumen seeds the spleen and liver. Our data show that the ∆*iscR* mutant is defective in growth in the intestinal lumen, implying that it would not be able to then seed the spleen and liver. In contrast, the *lcrF*^pNull^ mutant colonizes the intestine but not the spleen and liver, suggesting that, while IscR is required for growth in the intestine independent of the T3SS, IscR control of the T3SS is specifically required for *Yersinia* to successfully transition from a localized gut infection to disseminated infection of the hepatosplenic compartment.

The idea that IscR control of the T3SS in *Y. pestis* is a vestige of its evolution from an enteric pathogen is a very interesting one. **Our model is as follows.** When *Y. pseudotuberculosis* is ingested by a mammalian host, the shift in temperature removes thermo-repression of LcrF. However, *Yersinia* does not require the T3SS to grow in the intestine, so low levels of IscR under the low oxygen, low iron conditions keeps the T3SS to a minimum. In fact, we have preliminary data, which is part of another study, that it is important for *Y. pseudotuberculosis* to repress T3SS expression in the gut and that this is achieved by IscR. **Upon crossing the intestinal barrier, when oxygen tension is expected to increase, IscR levels are expected to rise along with T3SS expression, ready for the hepatosplenic environment where the T3SS is essential for bacterial growth.**

*Yersinia* IscR and the IscR binding site within the *lcrF* promoter are identical between *Y. pestis* and *Y. pseudotuberculosis*. This suggests that IscR control of LcrF pre-dates the development of *Y. pestis* from *Y. pseudotuberculosis*. The *Y. pestis* T3SS is not expected to be expressed in the flea, as the temperature is lower than 37˚C. When *Y. pestis* enters the mammalian host via a flea bite and enters the draining lymph node, it experiences conditions that are conducive to IscR, LcrF, and T3SS expression (anaerobiosis and iron depletion). Therefore, it is possible that when *Y. pestis* developed its flea-to-mammal route of transmission, IscR control of LcrF was already suited to this new life cycle.

We have included a discussion of the implications of our data on evolution of pathogenic *Yersinia* in our revised manuscript (lines 43-44, 59-62, 426-436).

• Anatomically, where you would expect the system to be expressed, given the in vitro results presented here? It was unclear which tissues are predicted to have low or higher iron, and which would have low or higher oxygen levels. It would be helpful to mention that culture conditions are meant to mimic specific tissue sites (low iron, aerobic conditions are expected to mimic deep tissues, etc), to help better explain the expected and observed results in the oral infection model.

While we do not have a complete enough understanding of the conditions *Yersinia* experiences in different tissues within the host to clearly compare our in vitro conditions to a particular tissue (sugar sources, oxygen tension, etc), we have clarified how our results might apply to *Yersinia* infection (lines 40-41, 52-54, 154-156, 400-416, 450-452). Please also see the response to the comment above.

• The introduction focuses on iron homeostasis, iron availability, and iron-dependent regulation of virulence factors, but doesn’t mention what is known about oxygen-dependent regulation in Yersinia or other bacterial species. A paragraph about oxygen-dependent regulation of virulence factor expression, and specifically the T3SS, is needed to help determine the gaps in this literature, and how this study addresses those gaps. An extended discussion about T3SS expression, and what is known about environmental triggers in general, would also be really helpful.

We thank the reviewer for this helpful comment. We have included a new paragraph on the impact of oxygen concentration on T3SS expression in several enteric pathogens (lines 103-123).

• The novelty of this study needs to be outlined more clearly. For example, lines 130-133 state that it is known IscR is essential for T3SS expression, is the link with iron availability already known? Or was this unclear prior to this study? What was known about oxygen-dependent regulation of T3SS in this bacterium, or in other bacterial species? Has there been a link between IscR-dependent T3SS expression and oxygen, or is this a novel aspect of this study?

We have significantly revised our abstract to make it clear that, while IscR was previously been shown to be important for type III secretion, “a direct regulatory role for iron and oxygen in *Yersinia* T3S has not been reported and it remains unknown why IscR evolved to control the *Yersinia* T3SS.” Our study **(1)** demonstrates the effect of iron and oxygen on the *Yersinia* T3SS (which has not been previously shown), **(2)** delineates the mechanism by which this occurs: Fe-S cluster demand directly controls levels of IscR, which in turn directly regulates LcrF, **(3)** suggests that *Yersinia* uses IscR to sense crossing the intestinal barrier for optimal T3SS expression during infection, and **(4)** shows, for the first time, that IscR is required for *Y. pestis* T3S and suggests that IscR control of the T3SS evolved in *Yersinia* prior to the split between *Y. pseudotuberculosis* and *Y. pestis*. We have bolstered discussion of these important points throughout the revised Abstract and Discussion sections.

• Densitometry is needed throughout to determine differences in protein levels relative to loading controls. This quantification was mentioned in the text describing Figure 6, but the quantification should be indicated on the blots throughout. This would also help with the interpretation for Figure 3, where it was difficult to determine if mRNA levels were different than protein levels.

Figures 3, 4, and 5 have been revised to include densitometry measurements.

• Changes in T3SS expression are described differently in the text than are shown in the figures; the figures show normalized data, while the text describes fold changes. These should match, and I think the fold changes are more helpful in visualizing induction.

We believe it is important to present the normalized data (as “raw” as possible) in the figures. The fold changes were included in the text of the manuscript to enhance discussion of the data.

• It was unclear why the authors thought anaerobic respiration would alter IscR-dependent phenotypes (Figure 5). The authors found the same result under these conditions, but it was unclear why this could have had an impact.

In EHEC, T3SS activity was found to be potentiated by respiration (Ando et al 2007 *Micro*). When we began our study, we therefore explored using several different terminal electron acceptors, but found that iron and oxygen controlled the *Yersinia* T3SS similarly regardless of the TEA acceptor used (or no TEA used). Therefore, we believe it is important to show type III secretion using both fermentative conditions and as well as anaerobic respiratory conditions. We have clarified this in our revised manuscript (lines 238-240).

• A recent Salmonella study showed that IscR regulates T3SS expression, although the conditions that promote expression (high iron, low oxygen) contrasted with the Yersinia conditions that promote T3SS expression (low iron, low oxygen). This recent study was mentioned at the end of the discussion, but should also have been mentioned in the introduction to this study, in addition to any evidence that may suggest the IscR-dependent regulation differs in the two species. Maybe the promoter binding motifs are slightly different? Maybe these systems have adapted differently for intracellular vs. extracellular growth?

This *Salmonella* IscR study was mentioned in the original Introduction, but we have added to this in the revised Introduction (lines 147-157).

Both the *Salmonella* IscR binding motif regulating the T3SS positive regulator HilD and the *Yersinia* IscR binding motif regulating the T3SS positive regulator LcrF are so-called type II motifs, that can be bound by both apo- and holo-IscR. The distinction is that IscR represses *hilD* in *Salmonella* and stimulates activation of *lcrF* transcription in *Yersinia*, most likely based on the relative location of the type II motifs to the promoter elements of the respective genes. This explanation has been added to our revised Discussion (lines 441-443).

Reviewer #2: No major issues

Reviewer #3: The IscR protein is conserved among enteric Gram-negative pathogens and the role of IscR as a sensor of Fe-S cluster demand, which depends on iron and oxygen has also been studied in Salmonella, Pseudomonas aeruginosa and E. coli, making the observation that IscR regulates T3SS in response to iron and oxygen less surprising. However, the overall outcome of IscR-mediated control on T3SS can be very different, indicating that this system co-evolved to adjust expression of these important virulence traits to the bacteria-specific colonized niches. The author should/could more emphasize the novelty of their study.

In our revised manuscript, we propose that IscR control of the *Yersinia pseudotuberculosis* T3SS allows the bacteria to sense when they have crossed the intestinal barrier and turn on T3SS expression (lines 40-41, 154-156, 400-402). This is in contrast to how IscR has been coopted in *Salmonella* to control the SPI-1 T3SS, but consistent with the enteropathogenic *Yersinia* infection cycle.

In addition, we have emphasized the implications of our findings on the infection cycles of both *Y. pseudotuberculosis* and *Y. pestis* as well as the evolution of *Y. pestis* from *Y. pseudotuberculosis* (lines 43-44, 59-62, 400-424, 426-436).

Overall the authors address a topic that is interesting for our understanding of the role of the Isc regulator family for virulence gene control. The data convincingly show that IscR binding on the lcrF promoter is critical for T3SS and depends on oxygen and iron availability, but the quality of some of the figures could be improved (for detailed comments see below).

Fig. 2B: The authors clearly showed that iscR expression is clearly induced at 37°C under aerobic conditions, but the regulatory mechanism remains unknown.

The regulatory mechanism is likely a result of derepression of holo-IscR on the *isc* promoter. Under aerobic conditions, levels of holo-IscR should decrease, leading to an increase in overall IscR levels, as shown in Figure 2B. We have included discussion of this in our revised manuscript, lines 351-368.

**Part III – Minor Issues: Editorial and Data Presentation Modifications**
Please use this section for editorial suggestions as well as relatively minor modifications of existing data that would enhance clarity.


Reviewer #1: Minor comments:
• Author Summary (lines 55-58): this described the varied environments enteropathogenic Yersinia would experience during infection, but what about Y. pestis? Does it also encounter an iron-replete, low oxygen environment like the intestine? This information could be included here, but could also be included at multiple points in the manuscript where comparisons between different Yersinia are made.

We have included a new paragraph in the Discussion (lines 426-436) that discusses the implications of our findings on *Y. pestis*, as well as throughout the manuscript (lines 43-44, 59-62).

• Lines 74-77: emphasize this is prior to infection.

We have revised this sentence as suggested (line 74).

• Line 81: is lipocalin-2 also expressed by the epithelium? Please include a citation here (line 82).

Citation now provided (line 83).

• Line 87-88: consider adding some specific examples of uptake systems.

This information was added. Please see lines 91-93 in the revised manuscript.

• Some of the information in the Introduction could be expanded to provide more specific examples: Line 90: which environments, which iron sources?

This information was added. Please see lines 91-93 in the revised manuscript.

• Line 95: which bacterial species?

This information was added (line 97).

• Line 96: which transcription factors?

This information was added (line 99).

• Line 116: which cellular processes?

The other cellular processes referred to were motility, adhesion to host cells, and hemolytic activity. This information was added (lines 134-136).

• Line 122-123: which Gram-negative organisms, which genes?

While the Introduction was reorganized for the revised manuscript, this information can now be found in lines (133-134).

• Lines 111-113: is it known which form of IscR binds to the lcrF promoter? Which motif is upstream of lcrF?

Lines 138-139 now explain that either holo- or apo-IscR can bind the type II motif in the *lcrF* promoter.

• Lines 326-327: Is this a gap that will be addressed in this study?

We are not clear on what the reviewer means here. Lines 326-327 included a sentence in the original Discussion section summarizing one of the findings of our study. We have revised the Abstract and Introduction sections to better clarify the gaps in knowledge addressed by our results.

• Line 127: which form of IscR?

We have included a discussion of how *Salmonella* IscR (either in the holo- or apo-form) binds a type II motif in the *hilD* promoter in the revised Discussion (Lines 439-443).

• Lines 149-155: It was unclear why the authors wanted to establish the growth phenotype of the lcrFpNull mutant.

We have added an additional sentence to explain the rationale for this experiment (lines 169-170).

• Line 186: typo, ‘p= 0.0 005’.

This error has been corrected (line 209).

• Line 234: typo, ‘amount’ should be ‘amounts’

This error has been corrected (line 258).

• Line 247-248: are there other expected alterations in the Apo-IscR strain?

As discussed in lines 271-272, this mutation leads to overexpression of IscR due to derepression of the *isc* promoter and a proton motive force defect under aerobic conditions.

• Line 371-374: Does the ∆iscR strain colonize Peyer’s patches? Does this suggest the T3SS is required to colonize all the listed tissues?

The T3SS is required for *Yersinia pseudotuberculosis* growth in some tissues at both early and late time points (spleen and liver), but not others (Peyer’s patches, mesenteric lymph nodes, and intestinal lumen). The ability of the *lcrF*^pNull^ mutant to colonize a given tissue reflects the requirement for the T3SS in that tissue, consistent with our overall model. However, the ∆*iscR* mutant is also defective for colonization of tissues where the T3SS is not a requirement for growth (ex-intestinal lumen and Peyer’s patches). This reflects the pleiotropic nature of the ∆*iscR* mutation. We have clarified this important point in the revised Discussion (lines 387-398).

• Line 393-396: this section was confusing, since it was mentioned earlier that the T3SS isn’t required for mesenteric lymph node colonization. Does this mean the system is expressed within lymph nodes, but isn’t needed?

The *Yersinia* T3SS contributes to colonization of the gut-associated lymph tissue two days post-inoculation but not at five days post-inoculation. The experiments that measured *Yersinia* gene expression in Peyer’s patches were performed at three days post-inoculation. Therefore, it seems that T3SS genes are expressed early during infection and contribute to early lymph tissue colonization, but immune responses in the lymph tissue later during infection may limit *Yersinia* growth regardless of T3SS activity. We have clarified the timing of gene expression measurements and T3SS requirement for virulence in lines 375-380, 406-408.

• Line 408: typo, ‘pneumonia’ should be ‘pneumoniae’

This term was deleted in the revised manuscript.

• Line 585-586: ‘Bases previously shown to be important for IscR binding are in bold’, the bases weren’t in bold.

This error has been corrected in the revised Figure 1.

• Figure 7: significance bar for Peyer’s patches needs to shift slightly to the right

This has been corrected in the revised figure.

Reviewer #2: Minor issues:
1. Line 206: Check coma in “p<0,0001”

This has been corrected in the revised manuscript (line 229).

2. Line 586; Figure 1: Does not appear that nts important for binding are not in bold as suggested in figure legend.

This error has been corrected in the revised Figure 1.

3. Line 605; Figure 2: Figure legend shows p value for ****, but there is only *** in the figure. Please make sure values match up.

This has been corrected in the revised manuscript.

4. Line 620; Figure 3: Please make sure p values in the figure legend match up with the figure.

This has been corrected in the revised manuscript.

5. Line 641; Figure 3: Please make sure p values in the figure legend match up with the figure.

This has been corrected in the revised manuscript.

6. Line 674: Please clarify if this is a representative of 5 independent experiments or combined data from 5 experiments.

We have clarified that the data shown in Figure 7 is combined from five independent experiments (line 709).

7. Figure 7: Please denote the limit of detection on each graph. Since your data is represented as CFU per gram, the limit of detection should be different for each tissue (the spleen will impact total volume more than the PP, and so will affect the primary dilution factor in the back calculation differently). Also, for samples below the limit of detection, it is more appropriate to set the CFU for these samples to the limit of detection for each tissue prior to calculating the mean and performing statistics.

We have revised Figure 7 to reflect the limit of detection per organ. This reanalysis did not alter any conclusions from these data.

Reviewer #3: l. 143: (12) should be (12)
l. 144: The unpublished observations should be more specified to illustrate that IscR is a global regulator

We apologize for the error. The sentence now reads “As IscR is a global regulator in *Yersinia* [24], the *lcrF*^pNull^ mutant should only be defective in T3SS control, while a ∆*iscR* mutant displays additional T3SS-independent phenotypes (see Figure 7 below).” (Lines 162-165)

The data in Figure 7 showing that the ∆*iscR* mutant is defective for intestinal colonization, but the *lcrF*^pNull^ mutant is not, demonstrates that IscR has additional effects on virulence independent of its effect on the T3SS.

l. 186: P=0.0 005 should be P=0.0005

This error has been corrected (line 209).

l. 248-253: This part seems contradictory or did I get this wrong? l. 249….the Apo-IscR mutant expressed LcrF, but was unable to secrete Yops due to a proton motive force defect….. l. 250 the Apo-IscR mutant had high IcsR levels..this correlated with high levels of LcrF and secreted YopE ….????

The Apo-IscR mutant is unable to secrete Yops due to a PMF defect under aerobic conditions (Miller et al 2014 *PLoS Pathogens*). However, we were surprised to find that the Apo-IscR mutant can secrete Yops under anaerobic conditions, either because the PMF is not required for T3S in the absence of oxygen or because the apo-IscR mutation does not cause a PMF defect in the absence of oxygen (lines 271-281).

Fig. 1A
An unspecific labelled DNA fragment or unlabeled competitive DNA would be helpful to distinguish between specific and unspecific binding. The presented gel shift assays are not fully convincing, as defined protein-complexes also appear with the lcrFPnull fragment.

We have repeated the EMSA experiment and have included this new data in the revised Figure 1. The data now clearly shows that IscR can bind the WT sequence but not the *lcrF*^pNull^ allele. In addition, cut vector backbone is present in the reaction and serves as a source of nonspecific DNA. We have clarified this point in lines 568-569.

Fig. 3 and Fig. 4
I suggest to add aerobic on top of Fig. 3 and anaerobic on top of Fig. 4 to make it easier for the reader to see which conditions were used.

We have added these titles to the figures.

Fig. 4A
The data presented demonstrate that even in the absence of the IscR protein, lcrF and yopE expression are upregulated in the absence of iron, although the overall level is reduced. Is the expression of other regulators, e.g. YmoA or RcsB influenced by iron under these conditions?

Transcript levels of *ymoA*, *rcsB*, and *cpxR* are all higher under iron limited compared to iron replete conditions in WT *Y. pseudotuberculosis* grown under anaerobic conditions (see below). However, this is also the case for the *gyrA* and *dnaK* housekeeping genes. This is likely due to the ability of the YopD T3SS secreted protein to regulate the RNA degradesome in response to secretion (Kusmierek et al 2019 *PLoS Pathogens*). We interpret this data to mean that increased IscR levels in response to iron limitation under anaerobic conditions increase LcrF levels and allow type III secretion to occur. As a result of YopD secretion under these conditions, mRNA levels of *lcrF* and other genes are stabilized, amplifying the difference between transcript levels in the -Fe/-O_2_ condition compared to +Fe/-O_2_ condition, where IscR levels are kept low. This idea that the YopD feedback loop affecting the RNA degradesome amplifies transcript differences is now discussed in lines 341-345).

To test this hypothesis, we used the Apo-IscR mutant that expresses high levels of IscR in both -Fe/-O_2_ and +Fe/-O_2_ conditions (see below). Indeed, we saw no difference in *ymoA*, *rcsB*, or *cpxR* mRNA levels for the Apo-IscR mutant ±Fe. These data suggest that iron controls the *Yersinia* T3SS independently of YmoA, RcsB, and CpxR.

Fig. 4B
Labelling of YopE and YopD need to be exchanged according to Fig. 3C.

This has been corrected.

Fig. 5 and 6
The different bands are not well defined due to different sizes and spillover of the slots and could be improved to exclude flow-over. In Fig. 6 it is further unclear what is loaded on lane 2? 5 lanes are visible, but only 4 are labelled. I assume the second lane was empty and the visible bands result from band 1 and 3.

For Figures 5 and 6, we have now used dashed lines to indicate lanes.

Fig. 8
The unspecific band just above the YopE band should be indicated as unspecific.

We have added a note to the Figure 8 legend and indicated the YopE-specific band in figure.
